# Early Identification and Eculizumab Treatment of Pregnancy-Associated Atypical Hemolytic Uremic Syndrome: A Case Report

**DOI:** 10.7759/cureus.91201

**Published:** 2025-08-28

**Authors:** Jianfang Wang, Yi Zhou, Qiulian Xu

**Affiliations:** 1 Department of Obstetrics, Jinhua People’s Hospital, Jinhua, CHN

**Keywords:** acute kidney injury, eculizumab, hellp syndrome complications, preeclampsia, pregnancy-associated atypical hemolytic uremic syndrome

## Abstract

Pregnancy-associated atypical hemolytic-uremic syndrome (p-aHUS) is a rare thrombotic microangiopathy that leads to increased fetal and maternal morbidity and mortality. Early identification and timely treatment can save lives and improve prognosis. p-aHUS should be highly suspected in patients presenting with microangiopathic hemolytic anemia, thrombocytopenia, and acute progressive renal failure in late pregnancy or postpartum. This case report describes a 26-year-old healthy pregnant woman who underwent an emergency cesarean section at 38 weeks of gestation due to severe preeclampsia and placental abruption. Postoperatively, she rapidly developed persistent hypertension, hemolysis, elevated liver enzymes, and thrombocytopenia, which were initially considered to indicate HELLP (Hemolysis, Elevated Liver enzymes, and Low Platelets) syndrome. However, the patient developed acute renal failure postoperatively, and her condition progressively worsened. After the diagnosis of p-aHUS was finally confirmed, the patient received plasma exchange and eculizumab treatment, resulting in significant clinical improvement.

## Introduction

Pregnancy-associated atypical hemolytic uremic syndrome (p-aHUS) is a rare but life-threatening form of thrombotic microangiopathy (TMA) that predominantly manifests during late pregnancy or the postpartum period [1,2]. Characterized by the triad of microangiopathic hemolytic anemia, thrombocytopenia, and acute kidney injury, p-aHUS is strongly associated with dysregulation of the alternative complement pathway, often triggered by pregnancy-related physiological stressors or complications such as preeclampsia, HELLP (Hemolysis, Elevated Liver enzymes, and Low Platelets) syndrome, or placental abnormalities [2,3]. The incidence is estimated at approximately 1 in 25,000 pregnancies, yet the associated maternal and fetal morbidity and mortality are substantial, with a high risk of progression to chronic kidney disease, end-stage renal disease, or even maternal death if not promptly recognized and treated [4,5].

The clinical presentation of p-aHUS often overlaps considerably with other pregnancy-related TMAs, particularly HELLP syndrome and thrombotic thrombocytopenic purpura (TTP), posing significant diagnostic challenges [3,6]. Both conditions may present with hypertension, proteinuria, hemolysis, thrombocytopenia, and varying degrees of organ dysfunction.

This case report describes a complex situation involving a healthy 26-year-old pregnant woman who developed p-aHUS rapidly after childbirth. The case is of particular clinical and academic value due to several unique features. First, it documents the acute postpartum onset of severe p-aHUS in an otherwise healthy young woman, initially misattributed to HELLP syndrome, a diagnostic pitfall frequently encountered in obstetric practice [3,6]. Second, the case details a comprehensive and prompt multi-modal therapeutic strategy, including early initiation of continuous renal replacement therapy, plasma exchange, and, crucially, eculizumab, a terminal complement inhibitor, despite the absence of identifiable complement gene mutations [1,2]. The successful use of eculizumab in this setting is especially notable given the limited data and experience with complement inhibition for p-aHUS in Asian populations, including China, where access and consensus guidelines remain limited [3,4]. Furthermore, the patient experienced rapid clinical and hematological recovery, normalization of renal function, and sustained remission after discontinuation of eculizumab, thus providing a valuable reference for the timing, efficacy, and safety of complement blockade in p-aHUS. We ensured the preservation of anonymity for the patient who provided informed consent.

## Case presentation

The patient is a 26-year-old healthy pregnant woman with no significant medical history, who attended regular prenatal check-ups. At 35+3 weeks of gestation, her blood pressure was recorded at 137/96 mmHg, and urine tests were negative. At 37 weeks, blood pressure measured at 103/69 mmHg, with urine showing 3+ protein. At 38+1 weeks, she presented to the hospital on November 5, 2024, at 19:51, due to 30 minutes of low back pain. On examination, her blood pressure was significantly elevated at 210/112 mmHg, with a non-stress test showing reactive fetal heart monitoring. An emergency ultrasound revealed a high echogenic mass approximately 85*70 mm on the right anterior wall of the placenta, poorly defined borders, and no significant blood flow signals, raising suspicion for placental abruption. The patient underwent an emergency cesarean section at 20:47 on November 5, delivering a live male infant weighing 2900 grams, with an Apgar score of 10 at 1 minute and 10 at five minutes. Intraoperatively, approximately one-third of the placenta was separated, with 200 ml of blood loss recorded. A urinary catheter was placed, draining clear urine.

At 1.5 hours after surgery, the patient reported severe headache and dizziness. Blood pressure was monitored at 189-198/96-101 mmHg, with laboratory feedback indicating hemolysis of blood samples. Five hours after surgery, the patient developed hyperkalemia. Seven hours after surgery, she exhibited pallor of the face and lips, with urine appearing dark brown and a volume of approximately 100 ml. Given the clinical picture of hemolysis, liver function abnormalities, and thrombocytopenia, HELLP syndrome was suspected, leading to transfer to the ICU for intensive management.

On postoperative day 1, the patient experienced persistent hypertension, significant thrombocytopenia (12 x 10^9/L), coagulopathy, acute kidney injury (creatinine 141.4 umol/L), oliguria (24-hour urine output of 150 ml), hyperkalemia (6.03 mmol/L), and liver dysfunction (ALT: 371.3 U/L) (Table 1). Continuous renal replacement therapy (CRRT) was initiated. By the third postoperative day, despite CRRT, the patient's creatinine levels continued to rise, accompanied by thrombocytopenia, a negative Coombs test, hemolysis, and fragmented RBCs (4%). Stool tests for infections returned negative, suggesting a diagnosis of atypical hemolytic uremic syndrome (aHUS). Testing for the von Willebrand factor-cleaving protease ADAMTS13 and genetic analysis were conducted, with recommendations for treatment with C3 or C5 complement inhibitors (e.g., eculizumab). Subsequent laboratory results indicated normal ADAMTS13 levels, with no mutations found in aHUS-related genes.

**Table 1:** Laboratory data obtained on the first and second days of hospitalization WBC: white blood cell; PLT: platelet; Hb: hemoglobin; Fib: fibrinogen; DD: d-dimer; PT: prothrombin time; APTT: activated partial thromboplastin time; ALT: alanine aminotransferase; AST: aspartate aminotransferase; Cr: creatinine; BUN: blood urea nitrogen; LDH: lactate dehydrogenase; TB: total bilirubin; DB: direct bilirubin.

Biochemical analysis	Day 1		Day 2		
	20:11	22:46	04:09	08:31	18:37
WBC（3.5-9.5 x 10^9/L ）	5.96	15.77	10.04	7.59	8.61
PLT（125-350 x 10^9/L）	139	50	58	12	59
Hb（115-150 g/l）	128	110	91	78	62
Fib（2.38-4.98 g/l）	2.8	1.01	0.86	1.60	2.35
DD（0-500 ng/mL）	2806.00	42637.00	42871.00	_	_
PT（9.4-12.5 S）	9.90	12.60	16.70	17.20	14.10
APTT（25.1-36.5 S）	28.30	24.80	37.70	39.70	46.90
Potassium（3.5-5.3 mmol/l）	4.21	6.24	4.40	6.03	3.50
ALT（7-40 U/L）	121.8	478.4	433.1	371.3	213.0
AST（13-35 U/L）	263.7	_	1136.8	1296.3	_
Cr（41-81 μmol/L）	79.4	104.4	120.6	141.4	200.1
BUN (2.6-8.5mmol/L)	5.52	6.58	7.19	8.77	9.52
LDH（120-250 U/L）	_	_	3804.6	4840	_
TB（0-21μmol/L）	5.8	32.3	53.0	89.7	113.9
DB(0-4μmol/L)	1.5	6.3	13.4	32.8	48.6

On postoperative day 4, the patient began undergoing plasma exchange, with a total of five sessions completed during her ICU stay (November 9, 10, 11, 12, and 13). By the eighth postoperative day, eculizumab therapy was initiated at a dosage of 900 mg IV weekly (administered on November 13, 20, and 27). Due to potential risks of fatal meningococcal infections associated with eculizumab, ceftriaxone (2.0 g IV daily) was administered for infection prophylaxis, and meningococcal vaccination was administered by the local health department.

**Figure 1:**
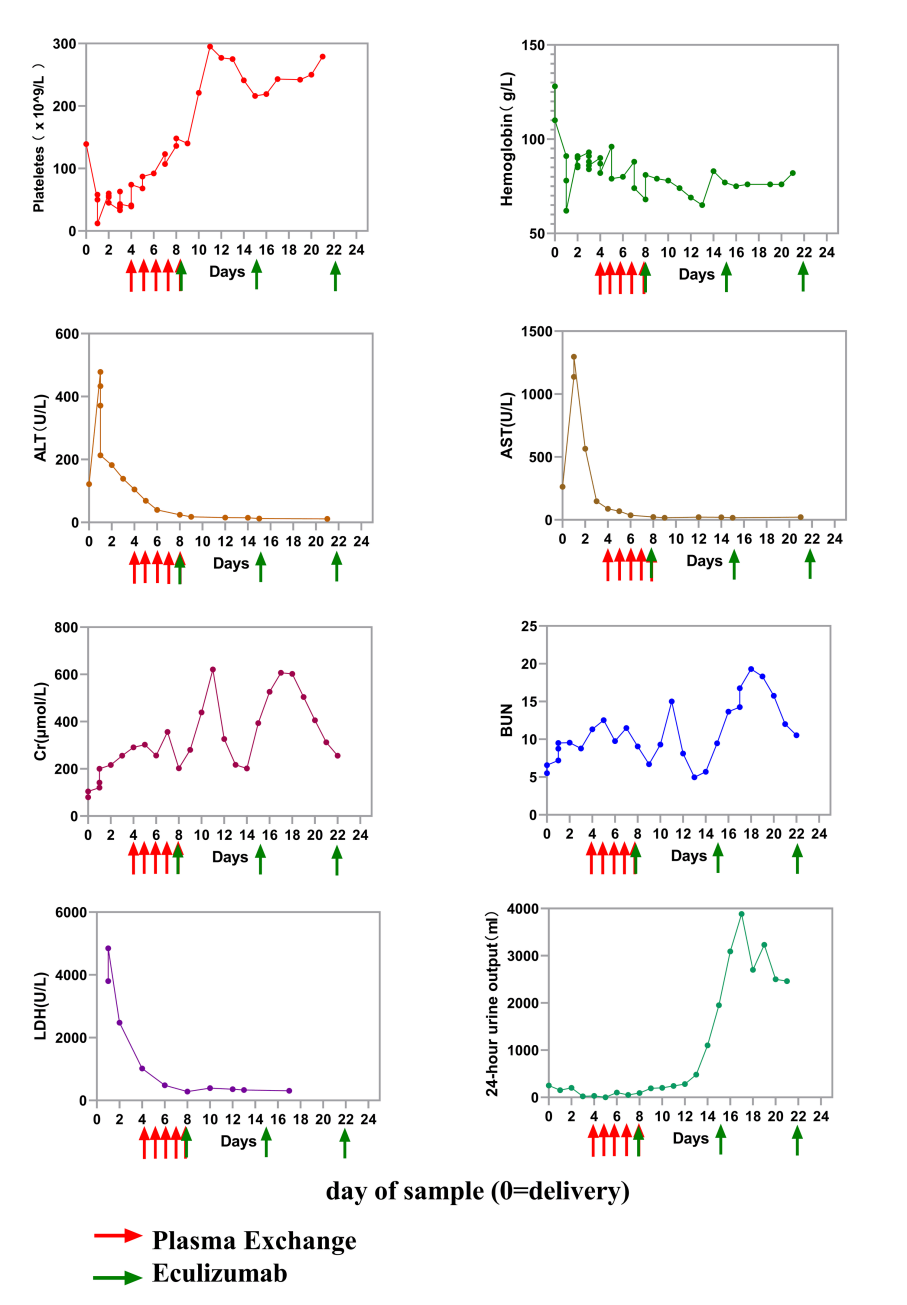
Development of selected laboratory values during the early stages of the pregnancy-associated atypical hemolytic uremic syndrome with administration of plasma exchange and eculizumab. In the 24-hour urine output plot, Day 0 shows the three-hour urine output of 250 ml. On the eighth day, superimposed red and green arrows were shown, indicating that the patient received both plasma exchange and eculizumab therapy on that day.

After treatment, the patient's urine output increased, and levels of blood urea nitrogen and creatinine rapidly declined (Figure 1). On postoperative day 22, she was discharged home. Following discharge, she continued outpatient management for p-aHUS with weekly monitoring of renal function and related parameters while receiving eculizumab (900 mg IV weekly on December 4, 11, and 27). At 52 days postpartum, her creatinine was 76.1 umol/L, hemoglobin was 106.00 g/L, renal function normalized, and urine tests showed no significant proteinuria or hematuria. Eculizumab treatment was discontinued, and she has remained relapse-free for the past three months.

## Discussion

This case emphasizes that when pregnant women have hypertension and related complications, p-aHUS should be considered as one of the differential diagnoses. Early diagnosis allows timely treatment measures, which can improve patient prognosis.

 In this case, the patient developed thrombocytopenia, elevated liver enzymes, elevated LDH, decreased hemoglobin, abnormal coagulation function, and soy sauce-colored urine after surgery. These symptoms progressed rapidly, leading to the patient being diagnosed with complete HELLP syndrome for the first time during this episode. After that, the patient's condition deteriorated rapidly; acute kidney injury developed, and creatinine continued to increase despite continuous renal replacement therapy (CRRT). Patients with HELLP syndrome usually improve within a few days after delivery and mainly present with liver damage rather than kidney damage. However, in this case, the patient's creatinine level progressively increased after delivery, and the disease lasted for more than 72 hours. Therefore, the diagnosis was more consistent with p-aHUS.

p-aHUS must still be distinguished from TTP. Although both TTP and p-aHUS are types of TMA, the distinct treatments required make TTP the primary diagnosis to rule out [7]. Typical manifestations of TTP include fever, mild renal insufficiency, severe thrombocytopenia, and neurological involvement [8]. Additionally, ADAMTS13 enzyme activity is usually less than 10% [9]. This patient exhibited neither neurological symptoms nor fever. The patient’s ADAMTS13 activity was normal, so TTP can be ruled out.

In addition, Shiga toxin-producing E. coli-associated hemolytic-uremic syndrome (STEC-HUS) occurs predominantly in children, accounting for up to 90% of cases. It is characterized by a clinical presentation with diarrhea and the detection of Shiga toxin-producing E. coli in stool samples [10-12]. Thus, the patient's negative stool test, including culture and PCR for Shiga toxin genes, makes this diagnosis unlikely.

The current international consensus on aHUS states that its clinical presentation and management are consistent, regardless of whether it is inherited, acquired, or of unknown etiology. The FDA has sanctioned the use of complement inhibitors, including eculizumab, as a proficient therapeutic option for managing aHUS [13-15]. For patients with TMA in whom TTP cannot be excluded, plasma exchange remains the first-line therapy. However, once clinicians exclude TTP, eculizumab is the best treatment [16,17]. Gupta et al. conducted a systematic review of English articles on aHUS during pregnancy or postpartum and found that the remission rate (88%) of patients with aHUS during their first pregnancy who received eculizumab treatment was significantly higher than that of patients who did not receive the treatment (57%, P = 0.02) [13]. Nonetheless, the specifics regarding the duration, dosage, and frequency of eculizumab administration remain ambiguous. Numerous studies have indicated that either reducing the dosage or completely stopping eculizumab therapy could result in a relapse of TMA, diminish the overall effectiveness of the treatment, and lead to a swift decline in the function of essential organs. Conversely, it has been proposed that eculizumab may be safely withdrawn after the attainment of clinical remission and the normalization of all hematological and renal dysfunctions, particularly in cases of aHUS where the initiating factor is distinctly recognizable [18]. Patients with p-aHUS experience persistent activation of the complement system and irreversible renal function damage. Although the condition may be effectively relieved in the short term, long-term treatment and follow-up remain necessary. Treatment should last at least 6 to 12 months, with renal function recovery taking at least three months [17].

The patient began plasma exchange on the fourth day after the operation, but the symptoms failed to improve. Eculizumab treatment was initiated on the ninth day after the operation. Although the treatment was initiated somewhat late and had some limitations, plasma exchange combined with eculizumab effectively controlled the condition. The patient’s pregnancy outcome was good, and she was discharged after improvement. The patient underwent regular follow-up in the nephrology department, and her renal function remained normal. After discharge, the patient underwent a check-up once a week. Eculizumab treatment was discontinued 52 days after delivery. No recurrence has been observed during the three months following treatment cessation.

## Conclusions

This case provides important insights into the management of p-aHUS, emphasizing the necessity for heightened clinical vigilance and a multidisciplinary approach in peripartum thrombotic microangiopathy. Our experience supports the growing evidence that early recognition of persistent microangiopathic hemolysis and renal dysfunction post-delivery, followed by prompt initiation of complement inhibition, can favorably alter the natural course of p-aHUS. Despite a negative genetic workup in our patient, the clinical response to eculizumab corroborates recent findings that complement dysregulation may be functionally relevant even in the absence of identifiable genetic mutations, suggesting a broader etiopathogenic spectrum than previously appreciated. This highlights the limitations of relying solely on genetic testing for diagnostic certainty and reinforces the role of empirical therapy in urgent clinical scenarios.

Nevertheless, several limitations must be acknowledged. The single-case nature of this report restricts the generalizability of our observations, and the relatively short follow-up period precludes definitive conclusions regarding long-term renal outcomes and relapse risk. In addition, the inability to delineate the precise molecular mechanism underlying complement activation in the absence of detected mutations leaves residual uncertainty regarding individualized risk stratification and recurrence prediction. These shortcomings underscore the need for more extensive, prospective studies in diverse populations to optimize diagnostic algorithms, refine treatment protocols, and elucidate the role of adjunctive therapies. As eculizumab access expands, ongoing evaluation of cost-effectiveness, optimal duration of therapy, and post-treatment surveillance strategies will be crucial, particularly in resource-limited settings. Ultimately, our case reinforces the imperative for early, protocolized intervention and highlights the evolving landscape of p-aHUS management, while advocating for continued research to address existing gaps in knowledge.

## References

[1] Winchester ML, Platzbecker R, McMahon M, Parrish M (2019). Eculizumab maintenance and the prevention of atypical hemolytic uremic syndrome relapse during pregnancy: a case report. J Med Cases.

[2] Domínguez-Vargas A, Ariño F, Silva D, González-Tórres HJ, Aroca-Martinez G, Egea E, Musso CG (2024). Pregnancy-associated atypical hemolytic uremic syndrome: a case report with MCP gene mutation and successful eculizumab treatment. AJP Rep.

[3] Hu C, Zhang P, Xu Q (2025). CFH nonsense mutation-mediated pregnancy-associated atypical hemolytic uremic syndrome: case report. Mol Immunol.

[4] Meena P, Gala R, Das RR (2025). Kidney and pregnancy outcomes in pregnancy-associated atypical hemolytic uremic syndrome: a systematic review and meta-analysis. Medicine (Baltimore).

[5] Fakhouri F, Roumenina L, Provot F (2010). Pregnancy-associated hemolytic uremic syndrome revisited in the era of complement gene mutations. J Am Soc Nephrol.

[6] Saad AF, Roman J, Wyble A, Pacheco LD (2016). Pregnancy-associated atypical hemolytic-uremic syndrome. AJP Rep.

[7] Azoulay E, Knoebl P, Garnacho-Montero J (2017). Expert statements on the standard of care in critically ill adult patients with atypical hemolytic uremic syndrome. Chest.

[8] Zini G, De Cristofaro R (2019). Diagnostic testing for differential diagnosis in thrombotic microangiopathies. Turk J Haematol.

[9] Sarno L, Conca P, Capuano A, Tarantino G, Russo D, Guida M (2022). A life-threating postpartum atypical hemolytic-uremic syndrome with multiorgan involvement. J Clin Med.

[10] Shen YM (2016). Clinical evaluation of thrombotic microangiopathy: identification of patients with suspected atypical hemolytic uremic syndrome. Thromb J.

[11] Yenerel MN (2014). Atypical hemolytic uremic syndrome: differential diagnosis from TTP/HUS and management. Turk J Haematol.

[12] Asif A, Nayer A, Haas CS (2017). Atypical hemolytic uremic syndrome in the setting of complement-amplifying conditions: case reports and a review of the evidence for treatment with eculizumab. J Nephrol.

[13] Gupta M, Govindappagari S, Burwick RM (2020). Pregnancy-associated atypical hemolytic uremic syndrome: a systematic review. Obstet Gynecol.

[14] Noris M, Mescia F, Remuzzi G (2012). STEC-HUS, atypical HUS and TTP are all diseases of complement activation. Nat Rev Nephrol.

[15] Gupta M, Feinberg BB, Burwick RM (2018). Thrombotic microangiopathies of pregnancy: differential diagnosis. Pregnancy Hypertens.

[16] Sheerin NS, Glover E (2019). Haemolytic uremic syndrome: diagnosis and management. F1000Res.

[17] Ávila A, Cao M, Espinosa M, Manrique J, Morales E (2023). Recommendations for the individualised management of atypical hemolytic uremic syndrome in adults. Front Med (Lausanne).

[18] Kumar D, King M, Jim B, Acharya A (2019). Recurrent case of pregnancy-induced atypical haemolytic uremic syndrome (P-aHUS). BMJ Case Rep.

